# Influence of Form Factor on Microstructural, Mechanical and Electrical Properties of Electrically Conductive Polyvinylidene Fluoride Processed by Arburg Plastic Freeforming

**DOI:** 10.3390/polym18030353

**Published:** 2026-01-28

**Authors:** Nurettin Arikan, Kevin Klier, Ibrahim Mutlu, Michael Hartung, Yavuz Emre Yagci, Mustafa Ozgur Bora, Hans-Peter Heim

**Affiliations:** 1 Department of Biomedical Engineering, Kocaeli University, Kocaeli Universitesi Umuttepe Yerleskesi, 41001 Kocaeli, Türkiye; ibrahim.mutlu@kocaeli.edu.tr; 2 Farplas Otomotiv A.S., Taysad Organize Sanayi Bölgesi (TOSB), 41420 Kocaeli, Türkiye; e.yagci@farplas.com; 3 Institute of Material Engineering, Polymer Engineering, University of Kassel, Moenchebergstr. 3, 34125 Kassel, Germany; klier@uni-kassel.de (K.K.); hartung@uni-kassel.de (M.H.); heim@uni-kassel.de (H.-P.H.); 4 Department of Aircraft Airframe and Engine Maintenance, Kocaeli University, Kocaeli Universitesi Arslanbey Kampusu, 41285 Kocaeli, Türkiye; ozgur.bora@kocaeli.edu.tr

**Keywords:** additive manufacturing, Arburg Plastic Freeforming, electroactive polymer, piezoelectric polymer, PVDF, X-ray computed microtomography

## Abstract

The utilization of polymer-based additive manufacturing processes for the production of functional components, consumer goods, spare parts, etc., has increased thanks to recent technological advances. The Arburg Plastic Freeforming (APF) process is a promising AM technology, in which standard plastic granules are deployed, and droplets are discharged along a track instead of using continuously extruded straws, unlike other filament-based processes, to the benefit of various industries that require good mechanical properties while maintaining dimensional precision. Due to the round shape of the droplets and tracks, however, defects such as voids can occur between individual paths during processing, which affect, most notably, mechanical properties. The electrical/ferroelectric properties of conductive/electroactive polymers are also affected. This study focuses on determining the optimal form factor for processing a special grade polyvinylidene fluoride (PVDF) material whilst other parameters, along with the ones ascertained in previous work, are kept constant. Along with tensile tests, X-ray computed microtomography (µ-CT) and scanning electron microscopy (SEM) analyses are implemented, particularly to observe microstructural porosity. Electrical properties and possible piezoelectric behavior are investigated via an originally adapted analytical method. The results provide important insights into the APF process and printing high-performance plastics with individual features, expanding the potential for further applications.

## 1. Introduction

Additive manufacturing (AM) technologies have profoundly altered industrial production in recent times, and they are increasingly being used to produce end-use parts, i.e., products, in addition to rapid tooling and rapid prototyping [1,2,3,4,5]. These processes facilitate the development of innovative design and implementation of components by eliminating several limitations that traditional technological methods have [6,7].

In various industries, including the biomedical, automotive and aerospace industries, AM processes have been perpetually deployed [8,9,10]. Thermoplastic polymers have chiefly been worked up by the fused deposition modeling (FDM) process, one of the most common AM technologies [11]. The FDM process is cost-effective, easy to deploy, and flexible in terms of usability [12,13]. However, the production of filaments represents an additional phase in the manufacturing cycle, whereas using this kind of feedstock might also induce several disadvantages, such as subtlety of part quality related to filament diameter, stiffness, etc. [14,15], in addition to possible defects in the part structure, thus delivering inferior mechanical properties as compared to injection-molded parts [16,17].

The direct processing of pellet feedstock, as used in injection molding, has recently been engineered to overcome such limitations and to expedite the production, hence being called “fused granulate fabrication” (FGF) [18,19]; nevertheless, the weaknesses conferred by the FDM method have not been reduced much since, in both processes, continuous strands are laid down after extrusion and melting, and hence the adhesion between layers (also between the perimeters and infill) is purely thermal and often weak, and it is not yet independent from the material used, even when these polymers are deployed as structural adhesives [20].

A more promising and relatively new process, called Arburg Plastic Freeforming (APF), has been used to bridge the gap between rapid prototyping/tooling and mass production (here, injection molding) using qualified raw materials to achieve denser, stronger parts than FDM/FGF processes since its launch in 2013 [21]. It is designed to facilitate manufacturing for a broad range of thermoplastic materials that have already been processed by injection molding for years [22,23].

**Figure 1:**
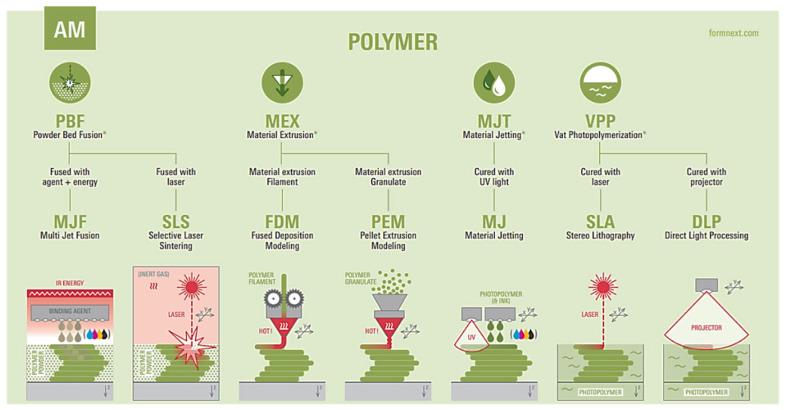
Additive manufacturing processes for polymers [25] (the graphics were created by Prof. Dr.-Ing. Steffen Ritter from Reutlingen University, Germany, in cooperation with Formnext/Mesago Messe Frankfurt GmbH, Frankfurt, Germany ©).

Although it is ever and anon categorized as a material jetting AM technology according to ISO/ASTM 52900 [24] considering the deposition of droplets, the APF process is also regarded in the material extrusion (MEX) subcategory [25], including, among others, pellet extrusion modeling (PEM); the feedstock polymeric material is melted and pressurized by a heated reciprocating screw, the droplets are then successionally deposited layer by layer using a nozzle that is controlled by a piezoelectric actuator [26]. Figure 1 summarizes the available AM processes for polymers.

The open material system of the APF process allows for the use of a wide spectrum of standard and engineering thermoplastics, including those certified for medical applications. In addition, depending on the specific machine configuration, the presence of multiple discharge units enables the additive manufacturing of multi-material parts with distinct physical characteristics. In the literature the versatility of this technology was highlighted by Spiller and Fleischer in a way that shows how the process can be used to produce metal parts; furthermore, a comparative analysis by Pinter et al. revealed that APF yields parts with a higher density and superior quasi-static mechanical properties compared to FDM. While these studies validate some of the benefits of APF, the issue of process accuracy is overlooked to some extent, unlike other thermoplastic AM processes, where geometric, i.e., dimensional, fidelity is well-documented. For industrial applicability, this is crucial, although this aspect of APF remains under-investigated, which represents a significant gap regarding the practical utility of the process. Eisele et al. have, indeed, reported several factors that concurrently influence [27] the APF process for acrylonitrile butadiene styrene (ABS), one of the most widely used thermoplastics. A characteristic process parameter, called the form factor (*FF*) or shape factor [28], also referred to as the drop aspect ratio (DAR) [27,29], becomes apparent, as it is used to vary the distance between single droplets and between droplet chains, thus affecting component density, strength, and even dimensional accuracy [30]. Thanks to advancements in imaging techniques as well as digital image processing, it is possible to validate these features of APF-processed parts, among others, by characterizing microstructures and observing porosity by X-ray computed microtomography (µ-CT) [27,31]; since the original physical object is not destroyed, numerous fields, including medical imaging, industrial computed tomography, natural sciences, stereoscopy, and many more have adopted this method for different applications.

**Figure 2:**
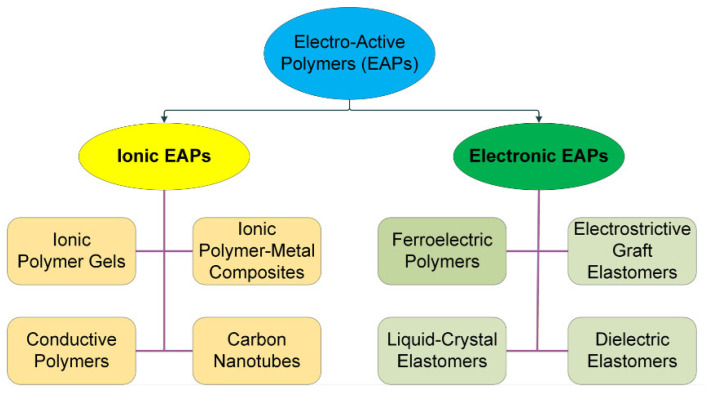
Classification of electroactive polymers.

Electro-active polymers (EAPs), particularly ferroelectric polymers and nanocomposites based on polyvinylidene fluoride (PVDF) matrix, have recently attracted great attention supposing that these materials will exhibit superb endurance, higher dielectric constant, low dielectric loss, and piezoelectric effect while offering a good deal of flexibility in a wide operating temperature range [32,33,34]. PVDF and its copolymers with trifluoroethylene (TrFE) and tetraflouoroethylene (TFE) have also been reported to exhibit piezoelectric behavior [35,36], while the addition, i.e., reinforcement of additives such as electroconductive carbon black, graphene, or multi-walled carbon nanotubes (MWCNTs) into its matrix will place the material in the nanocomposite material class. In Figure 2 the EAPs are categorized with regard to their mechanism of electroactivity [37].

Piezoelectric composites such as PVDF/BaTiO_3_, PVDF/MWNTs/BaTiO_3_ and so forth have also been studied to be developed and deployed as nanogenerators in energy harvesting as well as sensor applications [38,39,40,41,42]; indeed, the addition of ceramic particles might cause limitations, such as the interruption of 3D printing due to clogging, layer shifting, etc. [43]. With this in mind, reinforcing carbon-based additives is considered a better approach for (to-be-processed via additive manufacturing) PVDF, its copolymers, and PVDF-based composites. As piezo-ceramics are much denser than polymers and carbon, and their specific weight values are—in the same way—considerably higher (*ρ*_BaTiO3_ = 6.02 g/cm^3^) than those of the latter (*ρ*_C_ = 1.8–2.1 g/cm^3^) [43,44,45], it might be crucial to prefer carbon black or CNTs as reinforcement, i.e., additive in the polymer matrix to promote lightweight construction for, e.g., automotive, biomedical and any related applications. Also intended for the smart wearable device field, carbon black is reported to significantly enhance the crystallinity (thus—supposedly—dimensional accuracy), dipolar orientation, and piezoelectric performance of self-polarized energy harvesting materials [46,47], even considering that a better electrical conductivity can contribute to these features as related to percolation theory [47,48,49,50].

**Figure 3:**
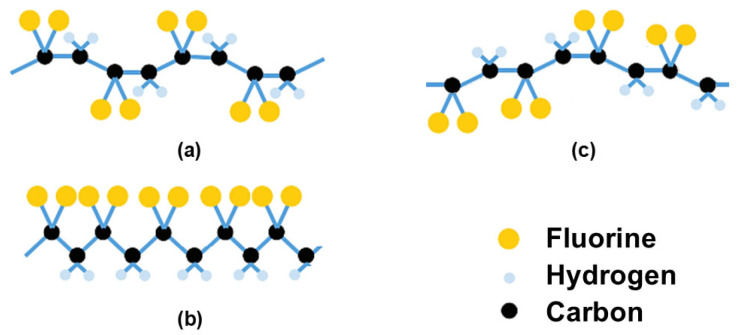
Molecular structure of α (**a**), β (**b**), and γ (**c**) phases of PVDF (derived from [53,57,58]).

Different from the prevailing yet nonpolar crystalline phase (α), occurring in trans-gauche-trans-gauche (TGTG) formation, the polar β-phase of PVDF forms a planar zigzag (TT) where trans bonds are represented by T’s that remain in the same plane as the carbon backbone [51]. Another phase “γ” is also polar and electroactive, and has a trans gauche (T3GT3G′) configuration with a higher trans fraction; nevertheless, this phase is stated as an uncommon state between α and β, and its polarization effect is weaker [52,53,54,55]. Based on existing literature, the β phase is achieved by mechanically deforming (e.g., stretching) PVDF near its melting point; this phase has an all-trans chain conformation where the fluorine (F) atoms are positioned on one side, and hydrogen atoms are located on the other side of polymer chain [56]. An illustration is given in Figure 3 to show the typical formation of these phases.

Since the β-phase is exclusively required to rebound the piezoelectric properties to PVDF [59,60], researchers have tackled the task of developing several methods for obtaining the β phase, including electrical [38,57] as well as thermal contact poling [58,61], milling [51,62] and so on, whereas it is suggested that there are other ways to yield it [38,61,63,64]. Self-polarized energy harvesting PVDF homopolymers and composites are in this regard relatively new developments, and different deposition processes have recently been studied [46,58,62]. As material extrusion additive manufacturing processes involve mechanical stretching, not only during extrusion but also through nozzle and/or printing platform movements, it springs to mind that it may be possible to print functional layers of exclusive yet existent materials that are designed to serve as sensors, actuators, energy harvesting surfaces or components; with respect to their availability and advantages, AM technologies should facilitate the production of such units in a rapid, cost-effective and sustainable way with or without poling, post-processing, etc. Apart from the conventional methods, for instance melt spinning, solvent casting, and electrospinning; 3D-printing of PVDF nanocomposites/generators has already started a trend with reputable achievements [65,66,67,68,69,70], partially comparable to those reported by Zhao et al. [71] and by other research groups [39,72,73].

Nevertheless, an important parameter called Curie temperature (*T_C_*) must be taken into account; materials typically show ferroelectric behavior below a certain phase transition temperature, known as Curie temperature. Above *T_C_*, the spontaneous polarization disappears [74] and the ferroelectric crystal transforms into the paraelectric state, causing the ferroelectric material to also lose its pyroelectric properties completely, since the paraelectric phase has a centrosymmetric crystal structure [75]. For PVDF, *T_C_* is often reported to be in the range of 195–198 °C [52,55,76], at a value above the melting point (*T_M_*) of the pure material [77], whereas its copolymers such as P(VDF-TrFE) are known to have a lower *T_M_* and *T_C_* [78,79,80] pertaining to the ferroelectric-piezoelectric (FE-PE) transition. Furthermore, applied research has found a *T_C_* of 103–110 °C [49,74,81], which should be associated with the loss of the piezoelectric effect (depolarization), hence not necessarily with the true FE–PE transition; in a way supporting this Sherman et al. have demonstrated that short exposure to temperatures up to 100 °C has no long-term negative effects on the piezoelectric performance of PVDF under normal circumstances [82]. Vasic et al. have also mentioned in their work [55] the phase transitions of polarized PVDF films, where melt-casted films, i.e., sensors, are reported to lose their piezoelectric effect at 110 °C (while the aging starts at 80 °C [83]) with regard to annealing processes and their influence [84,85].

In this study, mechanical and electrical properties of additive manufactured PVDF parts with different geometries by APF method are investigated in order to find the best-suited form factor. The processed material is a special grade of PVDF and electrically conductive thanks to its carbon-based additive, and rather than processing this material by FDM, APF is preferred thanks to its innovative working principle as well as the advantages of printed parts over those manufactured by FDM such as more densely packed structure and dimensional accuracy. On top of this, it is believed that multi-material 3D printing is more applicable in the APF process via interlocking mechanisms than in FDM; therefore, it should be easier to implement PVDF layers as functional surface, i.e., sections, while another decent material such as thermoplastic polyurethane (TPU) serves as the main material of a tailor-made part, as for instance this grade of PVDF is expected to act as the sensor/energy harvesting, even the conductive component (rather than an electrostatic discharge-ESD material) where the other material is the insulating enclosure. To the best of our knowledge, this work presents the first endeavor to process this electrically conductive grade PVDF in the APF method, starting from the premise that the material is suitable for being spray-nozzled onto an appropriate surface (e.g., build plate) as stated in the recent patents [86,87]. In this context, related literature, including studies on FDM 3D-printing of different PVDF grades, copolymers and composites [38,88,89], has also shed light on conceptualization of our experimental setup and achievement of new findings.

The results highlight an interesting correlation between the form factor in the manufacturing process and the residual porosity of the printed parts, thus affecting mechanical and electrical properties. An analytical model is also derived for a better monitoring of piezoelectric behavior of the material. Finally, for the optimization of related parameter sets and specifying the optimal degree of filling for intended applications, possible compromises are discussed.

## 2. Materials and Methods

### 2.1. Material Used

Arkema Kynar^®^ 340 polyvinylidene fluoride (PVDF) pellets provided by Arkema SA (La Défense, France) are used as feedstock. In addition to its melt processability, the material exhibits high chemical, abrasion, and thermal resistance in accordance with the manufacturer’s declaration [77]; this grade is also electrically conductive thanks to a special carbon-based additive. Some technical specifications of the material are given in Table 1.

The material in granulate form is let dry at 80 °C for 24 h to ensure smooth processing, although PVDF is reported as not hygroscopic (adsorbing less than 0.05% of water at room temperature) [56].

### 2.2. APF Process and Verification of the Material

**Figure 4:**
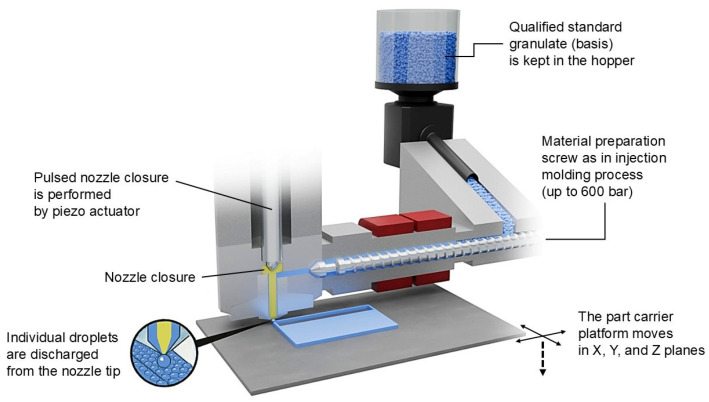
Schematic representation of the APF process.

Arburg Freeformer 300-3X HT “high-temperature” (ARBURG GmbH+CoKG, Lossburg, Germany) is used for additive manufacturing of the samples. As depicted in Figure 4, two heating zones can heat up the plasticizing cylinder with a 12 mm screw up to 350 °C. The plastification unit also includes a non-return valve. The thermoplastic material is fed into a discharge unit (under pressure control) after melting buffer is reached, and discharged in drop form via a piezo-controlled actuator with a maximum frequency of 300 Hz. The standard nozzle diameter is 0.2 mm, and layer-by-layer construction of the 3D print is executed by a component carrier table that can move in the X, Y and Z directions. As an open system machine, the Freeformer allows the operator to adjust all process parameters and set the drop shape and deposition, i.e., part properties. Thanks to its three separate discharge units, each with integrated material drying, the Freeformer 300-3X is able to manufacture multi-component functional parts such as hard-soft joints with a support structure. Its maximum build volume is 234 × 134 × 230 mm^3^ and the chamber can be heated up to 120 °C.

Since neither pure PVDF nor its reinforced grades have been previously processed by APF method, the verification of drop formation is carried out initially to determine optimal printing parameters. Droplet geometry is crucial for the quality of the printed parts as well as the stability of the process, and it can be adapted to the specified target layer thickness principally by altering temperature and discharge rate (DR) at the set temperature. Discharge rate can be defined as how much material is fed (delivered) from the nozzle per droplet. It is endorsed that the droplet height should always be slightly larger than the target layer thickness for good droplet bonding, i.e., layer adhesion, hence achieving a high component density. A ratio of approximately 25% to 30% is recommended by Arburg: as an example, if the target layer thickness is 0.20 mm, the droplet height may be set at 0.25 to 0.26 mm [96,97]. In fact, the form factor (*FF*) is principally the ratio of the droplet width (*W*) to the droplet height (*h’*), and it is also known as drop aspect ratio (DAR). *FF* is a characteristic process parameter, defined as

(1) $$FF=\frac{W}{h^{\prime }}\;\;\;\;\;$$

Droplet width (*W*) is measured perpendicular to the discharge direction, while droplet height (*h*’) is in the discharge direction. Therefore, the value calculated using Equation (1) is a theoretical form factor. Since the droplets are compressed, i.e., fused together during the process and subsequently a high component density is created, the droplet width must be longer than the layer thickness (*h*). As the droplets are fused, the droplet height (*h’*) ideally varies with the layer thickness (*h*). Hence, Equation (1) can be rearranged as follows, where *FF_init_* stands for initial form factor.

(2) $${FF}_{init}=\frac{W}{h}$$

As the droplets are pressed on the printing plate during the process, their width also changes due to gravity, and the true form factor (*FF_real_*) often differs from the default value (*FF_init_*). For preparation of data, one can utilize the droplet spacing (*W*), layer thickness (*h*), and form factor (*FF_real_*) using Equation (3).

(3) $$W=h\times {FF}_{real}$$

**Figure 5:**
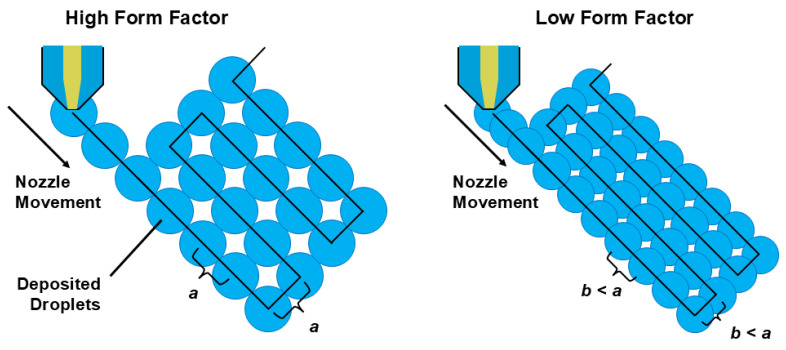
Representation of the process parameter form factor.

Nevertheless, the true form factor (*FF_real_*) must be determined through controlled experiments and visual inspection due to the fact that the true form factor affects the droplet spacing during nozzle movement (in the *XY* axis) and between printed layers (in the *Z* axis). By changing the true form factor, the infill level of the component, and therefore its density, can be significantly varied. The principal aim here is to achieve dimensional accuracy as well as good surface quality, and the highest possible component density. In Figure 5 a schematic description of the influence of the form factor is depicted.

**Figure 6:**
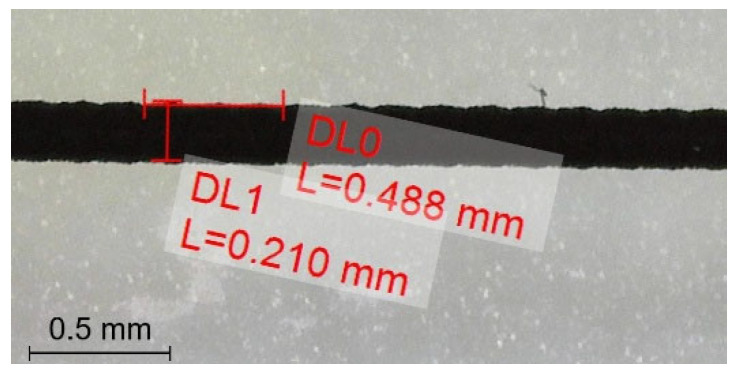
Initial straws for determining droplet dimensions. *DL0* represents *W* (albeit of three sequential droplets) and *DL1* represents *h*, respectively.

The parameters such as discharge rate, temperature of the heating zones and the nozzle (related to the viscosity of the material used), as well as chamber temperature, have a bearing on the form factor. In order to focus on the form factor exclusively, the others are kept constant: temperature zones are set at 220 °C and 235 °C, respectively, while the nozzle temperature is set at 255 °C with regard to the previous work [98,99,100], as well various experiences acquired during the fabrication of a filament for FDM process using this material as feedstock [101]. Chamber temperature is set at 90 °C to prevent warping of the samples, albeit attention is paid not to vary this value above 100 °C (considering the *T_C_* while the printed layers solidify). Frequency of piezoelectric actuator coefficient of printing is set to 65 Hz, then trial prints as material straws around 10 cm are acquired. These straws are optically examined using a light microscope, and subsequently images are captured. The length and height of drops are measured as shown in Figure 6.

Calculated *FF* values according to Equation (1) are 0.91 and 1.15, respectively, and considering the targeted layer thickness 0.2 mm, straws with *FF* = 0.91; 1.01; 1.11 are obtained. Besides being very dense and solid, the straws show sudden and very rapid cracking, i.e., fracture once pulled in both directions; this property is attributed to the brittle nature of the carbon additive. In this respect, *FF* values are decided to be increased for the samples to be printed, as explained in the following section.

### 2.3. Designing of Samples and Setting out APF Processing Parameters

Using ARBURG Freeformer software v2.31 for slicing the .stl files, the following samples are produced.

**Figure 7:**
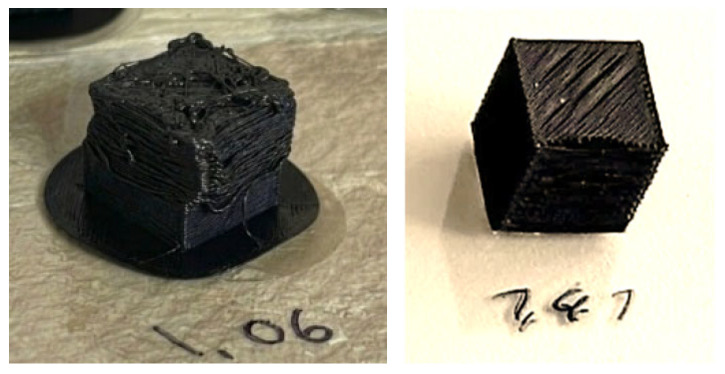
Deficient samples suffering from overfilling and layer shifting (left, *FF* = 1.06), and under-extrusion in another attempt (right, *FF* = 1.41). Samples with *FF* < 1.21 as well as *FF* > 1.31 show dimensional inaccuracy (e.g., sagittal length measured longer or shorter than 10 mm) due to the overfilling and/or overlapping, or contrarily because of under-extrusion for higher form factors, which is unpreferred for the end parts.

1. 10 × 10 × 10 mm^3^ calibration cubes, at least five of each, are printed in numerous form factors to determine the correct *FF* precisely, beginning with 1.06 to 1.41 and in intervals ranging 0.03 and 0.04 at coefficient value. Five different form factor values, from 1.16 to 1.31, are chosen for further investigation, since the initial visual results of samples clearly show that *FF* = 1.06, 1.09, and 1.11 lead to very overfilled shapes rather than cubes. Using a carbon steel Vernier caliper with 0–150 mm (0–6”) range, 0.02 mm resolution, and Metric/Inch dial, it is confirmed that dimensional inconsistencies up to 1.05 mm are measured. Images of two failed samples with *FF* = 1.06 and *FF* = 1.41, respectively, are given in Figure 7.

**Figure 8:**
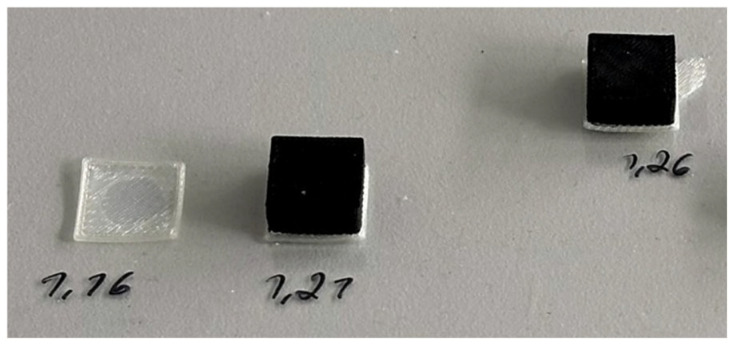
Warping phenomenon: Even a preliminary layer of support material is deposited before printing with PVDF material, causing either the part detaching from the plate or dimensional inaccuracy (independent from *FF*).

**Figure 9:**
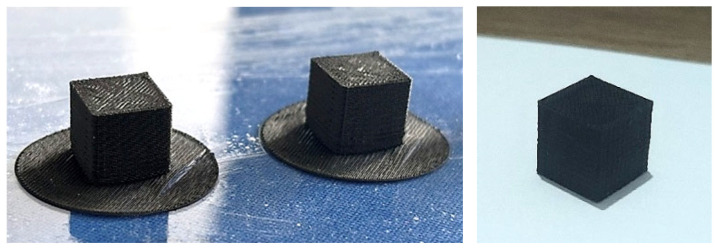
Successfully printed cubes with brims (**left**) that facilitated first layer adhesion, and after removing from the plate (**right**).

Samples with *FF* > 1.31 (1.34 up to 1.41) are intended not to be further investigated due to the fact that they show apparent defects such as under-extrusion, visible gaps between diagonal raster lines on the top layers, i.e., surfaces, etc. To avoid excessive warping (as to the semi-crystalline nature of PVDF), which has been encountered in previous studies regarding FDM process [98,99,101], the cubes are printed on smooth polycarbonate (PC) plates instead of textured ones. Furthermore, the warping could not be prevented, and another attempt was made to print the samples on the support material Armat 11 for better first layer adhesion; however, slight warping is still seen, as in Figure 8. Against this background, one-layer-brim is added to each sample’s perimeter, i.e., first layer in Shapr3D CAD (version 5.790) modeling software, then the exported .stl files are sliced once more; Magigoo Pro PC, an adhesive specifically designed for 3D printing compatible with not only PC, but also a broad range of polymers [102], is applied to the printing plates before placing it onto the printing platform of the Freeformer. The cubes are ultimately printed with success, displayed in Figure 9. The caliper is once more put to use in order to verify that the samples (with *FF* between 1.16 and 1.31) are dimensionally accurate with a margin of error of 0.12 mm maximum.

**Figure 10:**
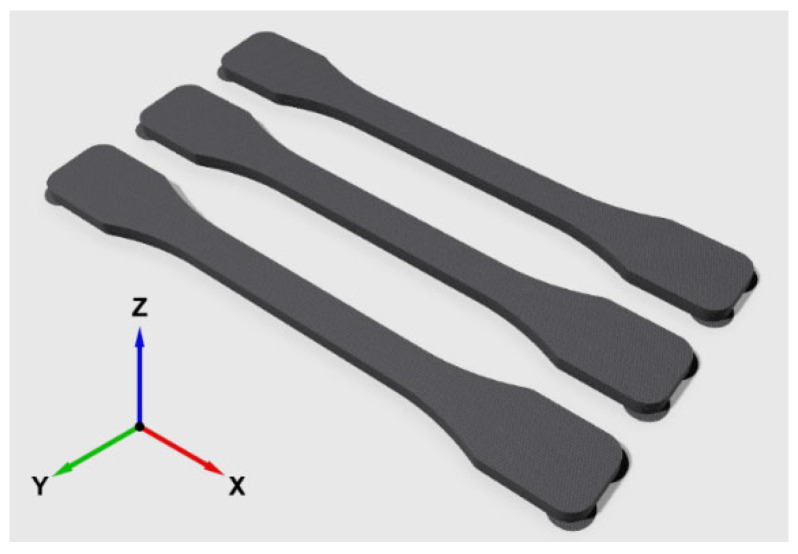
Placement of the tensile specimens on the printing table. Minimal brims known as mouse-ears are defined and applied for all corners of each sample in order to ensure the first layer adhesion, along with easy removal after printing so that the tensile test results are not affected.

**Figure 11:**
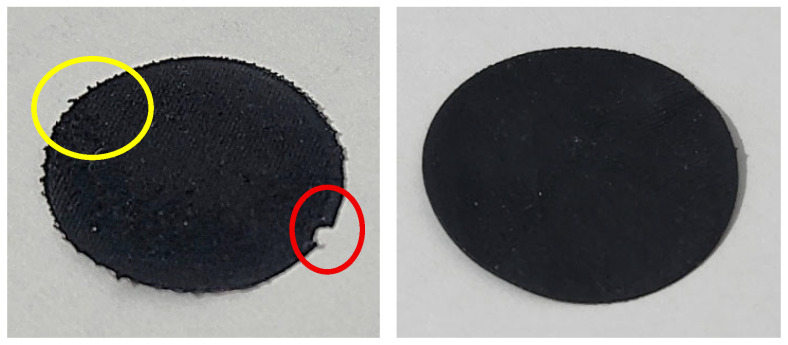
Circular single-layer samples with *FF* = 1.16 (**left**) and *FF* = 1.24 (**right**). Defects such as clumping due to overfilling (indicated by the yellow circle), as well as dimensional inaccuracy because of nozzle dragging (indicated by the red circle), are present along with the lower form factors, while higher *FF* values yield better parts, yet deteriorating as from *FF* = 1.28.

2. For tensile tests, 6 samples per each form factor are produced according to DIN EN ISO 527-2, Type 1B [103]. In order to ensure repeatability and reproducibility, and taking the build volume of the machine into account, slicing is performed as if 3 samples are placed flat on the printing platform (XY plane), actuating the Freeformer to produce the specimens with raster angles 45°, as illustrated in Figure 10.

3. Trusting that the material is adaptable to multi-material processing (with similar, i.e., compatible printing parameters) via interlocking mechanisms as mentioned in the introduction section, and in the hope that a single-layer part may rather function as a sensor owing to the ferroelectric/piezoelectric nature of PVDF, circular samples of one layer (with diameter of 29 mm and layer thickness of 0.2 mm) were produced. Partially as an example, one of the most common PVDF sensor/switches LDT0-028K from TE Connectivity [104] is taken into consideration since it possesses a piezoelectric PVDF polymer film as employed functional layer, and ⌀ = 29 mm is chosen to match the dimensions of the relevant part of the test device (explained in Section 2.7). For the samples with *FF* = 1.16 surface defects because probable nozzle dragging/plowing across the print are observed, while the samples with *FF* = 1.31 suffer from perspicuous under-extrusion, these are decided to be excluded from further investigation in the piezoelectric behavior tests (also due to the fact that such layers/components are not to be deployed as sensors or functional surfaces); samples with *FF* = 1.21, 1.24, and 1.28 are retained. In Figure 11, images of a defective and a proper sample are given.

**Figure 12:**
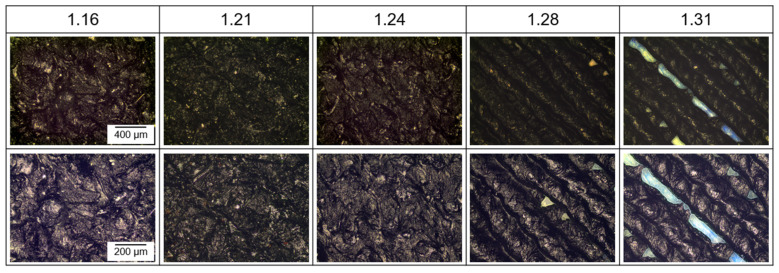
Surface images of circular single-layer samples with different form factors taken by light microscope (**top row**: magnified 5×, and **bottom row**: mag. 10×, respectively).

Figure 12 shows the surface images of these samples obtained by light microscope for a comparison.

As mentioned above, samples with *FF* = 1.16 and *FF* = 1.31 are excluded from further investigation because of the defects and lack of dimensional inaccuracy. Rasters related to nozzle movement can clearly be seen on the samples with *FF* = 1.28 and *FF* = 1.31, where micro-voids are also noticed, at an unacceptable level for the one whose *FF* is 1.31.

For all samples, the parameters except *FF* values are kept constant. Table 2 outlines which sample sets are produced and subsequently examined.

An overview of the machine parameters used for the Freeformer is given in Table 3.

### 2.4. Porosity Characterization by X-Ray Computed Microtomography (µ-CT)

X-ray microtomography (μ-CT) measurements are carried out using the Xradia 520 Versa provided by Carl Zeiss (Oberkochen, Germany). The X-ray source operated at a voltage of 80 kV and a power of 7 W. An objective with a 4× magnification and the LE1 filter is used with an exposure time of 5 s. The resulting voxel size is 4.955 μm. The graphical analysis of the results is performed using the software Avizo 9.4.0 by Thermo Fisher Scientific (Waltham, MA, USA).

### 2.5. Tensile Tests

Tensile tests are carried out at room temperature (23 ± 0.5 °C) using a universal testing machine UPM 1446 form ZwickRoell GmbH & Co. KG (Ulm, Germany). Test specimens, type 1B, are prepared according to DIN EN ISO 527-2 as described in Section 2.3. The tests are performed at a speed of 5 mm/min, and a preload of 5 N is applied prior to testing. Tensile strength and elongation at break are determined from five test specimens per form factor.

### 2.6. Investigating Fracture Surfaces by Scanning Electron Microscope (SEM)

**Figure 13:**
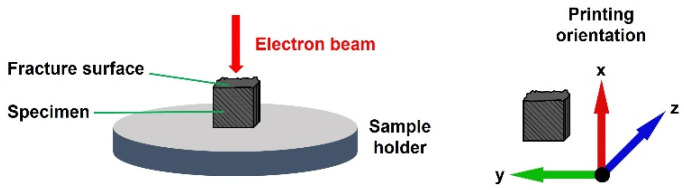
Sample placement for fracture surface investigation by SEM.

In addition, scanning electron microscopy (SEM) is used to examine the fracture surfaces of the samples. SEM images are acquired using a CamScan MV2000 microscope (Electron Optic Services Inc., Ottawa, ON, Canada) equipped with a secondary electron (SE) detector. The samples are mounted with the fracture surface facing upward and sputter-coated with gold prior to analysis. Images are recorded at magnifications of 100× and 1000× using an accelerating voltage of 10 kV. The fracture surfaces of samples produced with different form factors are compared. An illustration of the sample placement is given in Figure 13.

### 2.7. Investigation on Conductivity and Piezoelectric Behavior

Rohde & Schwarz GmbH & Co. KG (Munich, Germany; formerly HAMEG Instruments GmbH) HMC8012 Digital multimeter is used for both conductivity measurements and observing possible piezoelectric behavior.

**Figure 14:**
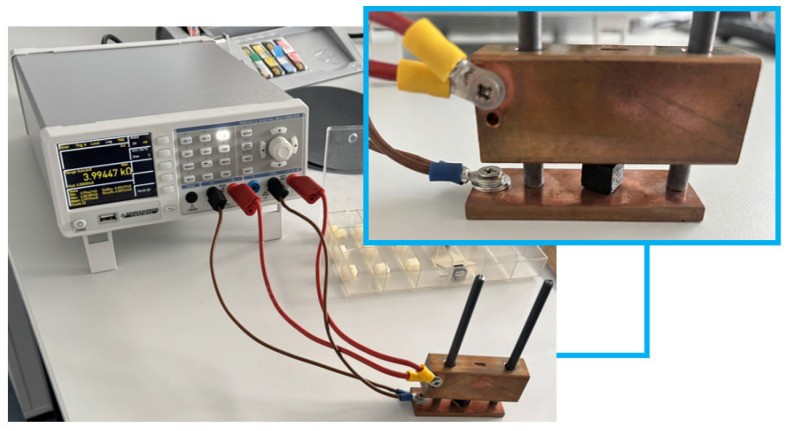
Digital multimeter and connected in-house developed apparatus for measuring electrical conductivity.

- Conductivity: Cubic samples are placed between the copper blocks of an in-house developed apparatus with attached electrodes as seen in Figure 14.

The noticed inconsistencies that initial measurements show are attributed to a partial lack of contact; therefore, the layers to be contacted are sandpapered gently to achieve an even surface, then a silver conductive paint (Doduco Contacts and Refining GmbH, Pforzheim, Germany) is applied by a dedicated paintbrush to each of them (other surfaces are not painted). Measurements are afterwards reiterated according to four-wire resistance measurement method in order to reduce the effect of test lead resistance, thus eliminating a possible voltage drop in the test leads [105]. Axial (XY) and cross-sectional (regarding both XZ and YZ) planes are recorded separately: two sets of values for each plane and sample with different form factor, gathered both instantaneously and within 1 min period, are recorded. Taking notice of the fact that the values are almost identical after numerous cycles—up to 5 min of continuous monitoring—the following Equation (4) is used to calculate the electrical resistivity of the specimens.

(4) $$\rho =R\frac{A}{l}$$

*ρ* represents here the electrical resistivity, where *R* is the electrical resistance of the specimen (the value read on the multimeter display); *A* is the cross-sectional area, and *l* is the length of the specimen, respectively. It is expressed by the SI unit ohm meter (Ω×m). For conductivity (σ) is the inverse of resistivity; calculations are performed using Equation (5) as follows.

(5) $$\sigma =\frac{1}{\rho }$$

Conductivity has the SI units of siemens per meter (S/m).

**Figure 15:**
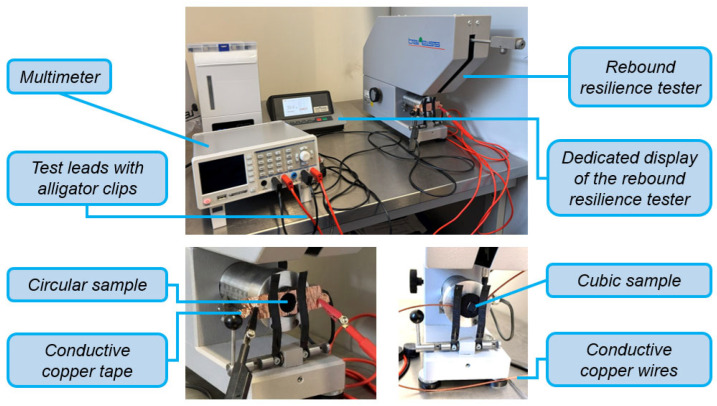
Experimental setup with multimeter and rebound resilience tester with Schob type pendulum (**top**), by which both circular single-layer (**bottom left**) and cubic (**bottom right**) samples are measured.

- Piezoelectric behavior: An experimental setup making use of both the multimeter and a rebound resilience tester with Schob-type pendulum (Bareiss Pruefgeraetebau GmbH, Oberdischingen, Germany) is arranged to carry out controlled measurements. Figure 15 represents the setup and installation details of specimens.

The conception is based on a case study, where the TE Connectivity LDT0-028K sensor is used as a flexible switch, and it generated an electric potential difference about 7 V after its tip was bent down, i.e., deflected approximately 2 mm; it is stated further on that voltages above 70 V could be generated if the bending is performed towards 90° [104]. To circumvent any inconsistencies associated with manual bending, the rebound resilience tester was employed to deliver standardized 0.5 J impacts. This energy level approximates the biomechanical force of a relaxed hand drop from 10 cm, if translated using the existing biomechanical models such as Zatsiorsky’s parameters [106,107], and aligns with the IK04 rating (IEC 62262) [108,109]. Although the equipment is intended for testing soft elastomers per DIN 53512 [110], ISO 4662 [111], and ASTM D7121 [112] standards, the cubic specimens and polyester-based sensor components ought to exhibit high hardness (Shore 71 D–87 D) [77,104,113]. Consequently, elastic deformation is negligible compared to soft materials (minimizing resilience effects), allowing the analysis to focus exclusively on the piezoelectric behavior of the PVDF. For a comprehensive comparison, measurements are performed firstly with the LDT0-028K sensor, afterwards implementing the single-layer samples with *FF* = 1.21, 1.24, and 1.28, respectively, and eventually with the cubes while paying attention not to apply impact to the silver-painted surfaces that are connected to the copper wires.

## 3. Results

### 3.1. Initial Determination of the Best Suited Form Factor

As stated in Section 2.3, the cubic samples are dimensionally accurate within a maximum margin of error of 0.12 mm. Aside from the number of failed samples, visible defects due to the overlapping of droplets (pertaining to the local clumping), or movement of the print platform (regarding the raster lines/gaps), etc., are observed less frequently when *FF* is set to 1.21 or 1.24. Therefore, the optimal form factor is considered to be 1.21 or 1.24, or somewhere in between. This can be supported by the fact that the tensile samples and circular single-layer specimens show similar, i.e., comparable flaws depending on the form factor with which the involved part is printed.

### 3.2. µ-CT

**Figure 16:**
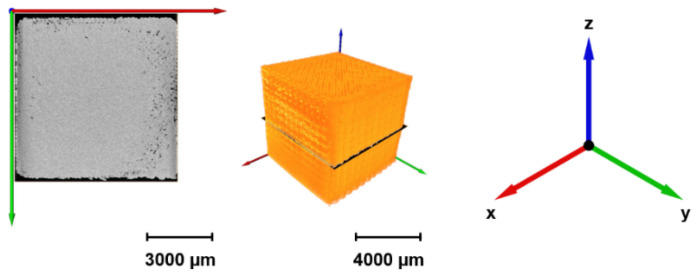
Reconstruction of the obtained projections on Avizo 9.4.0 software (here for *FF* = 1.24).

**Figure 17:**
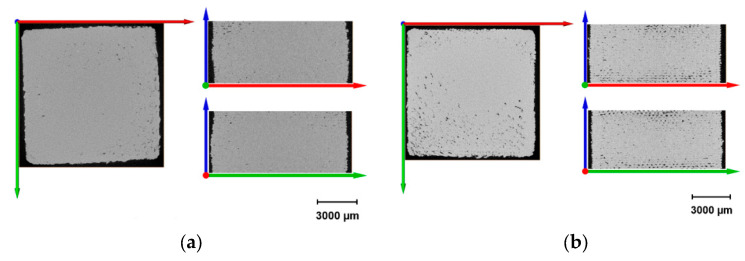

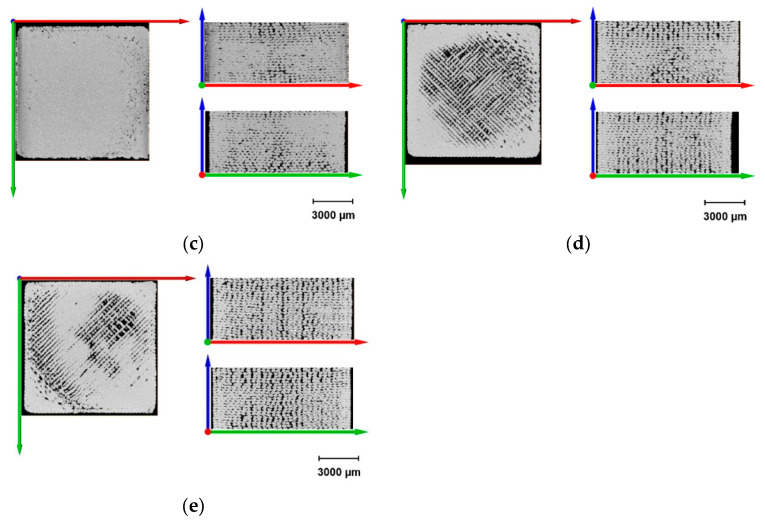
Reconstructed projections in axial, sagittal and coronal view of samples with different form factors: 1.16 (**a**), 1.21 (**b**), 1.24 (**c**), 1.28 (**d**), and 1.31 (**e**).

Slice images of the specimens with different *FF* values, acquired through adjustments after analyzing μ-CT results, are shown in Figure 16 and Figure 17.

There is a clear inverse correlation visible in the scans. As the form factor increases from 1.16 to 1.31, the porosity increases significantly, evolving from microscopic voids to structural gaps. *FF* = 1.16 is the most consolidated, whose top, i.e., axial view (XY plane) shows a nearly solid surface, indicating excellent material fusion. Defects seem to be minimal, the slight irregularities at the corners may have occurred because of clumping due to the acceleration/deceleration while printing, yet the central region is dense. The regional protrusion is, however, clearly visible in especially side scans (sagittal-XZ, and coronal-YZ views); thus, the dimensional accuracy is adversely affected. As one can see the sample is as if pressed from the top and overflowing to the sides. Aside from that, the results imply a near-fully dense part with porosity < 2%, and accordingly, the part is to exhibit a behavior close to an isotropic material whose properties are similar in all directions.

*FF* = 1.21 is assumed to be still dense regarding the axial view, yet both the sagittal (XZ) and coronal (YZ) scans reveal the onset of structural defects such as voids that may be considered as lack of fusion (LoF) voids. Regional protrusion is still present, however not as severe as that of *FF* = 1.16. In this regard, this form factor is also not favored where the dimensional accuracy is crucial for the end part.

As for *FF* = 1.24, local inter-droplet voids become clearly visible. Black gaps between beads may indicate that the overlap between adjacent raster lines was insufficient to close the gaps, hence increasing the anisotropy. Nevertheless, protrusion phenomenon fades almost completely away, therefore promoting dimensional accuracy. This shall be beneficial particularly for flat parts, since the Z-strength (considering interlayer bonding) may drop in some degree to the X/Y strength if the part height equals more than numerous layers.

*FF* = 1.28 shows in axial view a distinct cross-hatch (grid) pattern, though not taken as a whole. The solid infill seems to be partially broken down into a mesh which might indicate under-extrusion, i.e., imperfect deposition. Raster voids are no longer in microscopic scale, and these gaps may have been caused by the nozzle spacing while printing too wide for the amount of material deposited. Furthermore, large interconnected void networks are visible in the center. A significant reduction in mechanical performance is predicted, and the part will probably be susceptible to delamination under load. Finally, a slight concavity in the coronal scan is apparent that might be a sign of negatively affected dimensional accuracy, to the contrary of overfilled samples (*FF* = 1.16 and 1.21, respectively).

The axial view of *FF* = 1.31 depicts severe under-extrusion, where the diagonal raster lines have large parallel gaps running between them. This macroscopic porosity may lead to the lowest strength, and the structure is assumed to be highly permeable. Failure might likely occur via buckling of local/individual droplet clusters rather than bulk material yield.

### 3.3. Tensile Test Results

**Figure 18:**
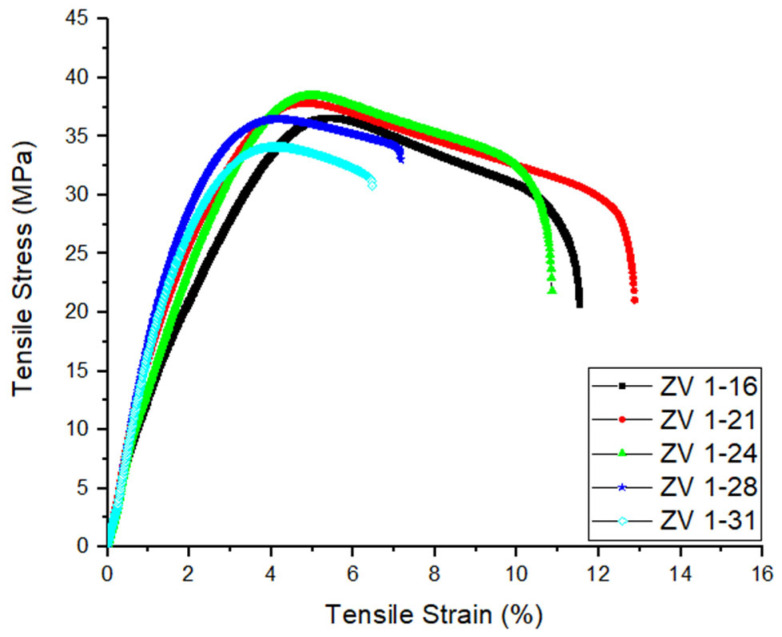
Tensile stress–strain curves of 3D-printed PVDF specimens fabricated with different form factors (from 1.16 to 1.31).

In Figure 18, tensile stress–strain curves of the samples with different form factors are given.

The PVDF specimens’ tensile stress–strain responses clearly depend on the form factor set during APF processing. Tensile strength and strain both peak at moderate filling levels (*FF* = 1.21 and 1.24), with corresponding strains of 12.87% and 10.86%, and strengths of 37.89 MPa and 38.66 MPa, respectively. These findings suggest that a small amount of overfilling improves the extruded layers’ overall consolidation, lowers the void content, and increases inter-droplet contact. Table 4 presents the relevant values in detail.

As previously documented for semicrystalline polymers like PVDF and polylactic acid (PLA), such advancements in interlayer fusion are known to improve load transfer efficiency and increase resistance to failure [114,115]. Mullaveettil et al. [116] observed that PVDF exhibits a distinctly elasto-plastic tensile response, with the shape and extent of the yielding zone being highly sensitive to the internal architecture and degree of bonding between adjacent extruded filaments in FDM process. Their results demonstrated that specimens with well-aligned and strongly fused filament paths show the highest tensile strength and strain due to improved load transfer and delayed crack propagation. This behavior is consistent with the present findings, where intermediate filled ratios (1.21–1.24) achieve optimal filament fusion and minimal void content. At overfilled levels (*FF* = 1.16), although the specimen still shows relatively high strength (36.46 MPa) and strain (11.51%), the mechanical performance is slightly inferior to that of the optimally filled samples. This implies that an overfilling limits stress transfer across filament borders by leaving a larger fraction of interstitial porosity and weaker interlayer diffusion. Low bead overlap leads to a heterogeneous density distribution, early micro void initiation, and reduced tensile characteristics, according to studies on FDM-processed polymers [117]. In contrast, when the *FF* value is increased further (1.28 and 1.31), both tensile strength (36.46 MPa and 34.12 MPa) and, more noticeably, strain (7.16% and 6.43%) show a clear reduction. This deterioration is likely linked to issues such as filament over-compression, the buildup of residual stresses, and deformation of the deposited bead geometry. At these levels, the extruded material is forced into the previous layers under elevated pressure, which can produce inconsistent bonding and localized internal stresses that act as early failure sites [118]. Moreover, in semicrystalline polymers like PVDF, excessive compression can disrupt the crystalline alignment that normally supports tensile deformation, making the material more prone to premature fracture.

### 3.4. SEM Characterization

**Figure 19:**
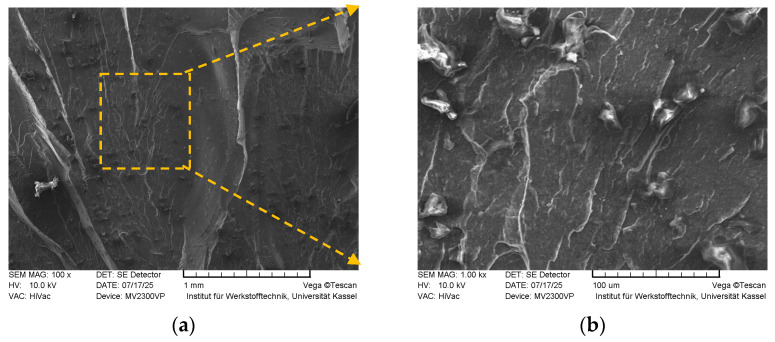
Fracture surface SEM images of the sample with *FF* = 1.16 (magnified 100× (**a**), and 1000× (**b**), respectively).

In Figure 19, fracture surfaces of the tensile sample with *FF* = 1.16 acquired by SEM (magnified 100× and 1000×, respectively) are given.

The fracture surfaces exhibit pronounced roughness, indicative of substantial plastic deformation. Prominent deep ridges, resembling tear ridges, are observed throughout the surface, which are characteristic of a ductile failure mode wherein the material undergoes yielding prior to fracture. These observations align well with the tensile test results, corroborating the material’s ability to sustain plastic deformation. Additionally, locally observed dimple-like arcs likely represent micro-void coalescence sites, which act to hinder mechanisms such as layer delamination, thereby contributing to the overall fracture resistance of the thermoplastic material.

**Figure 20:**
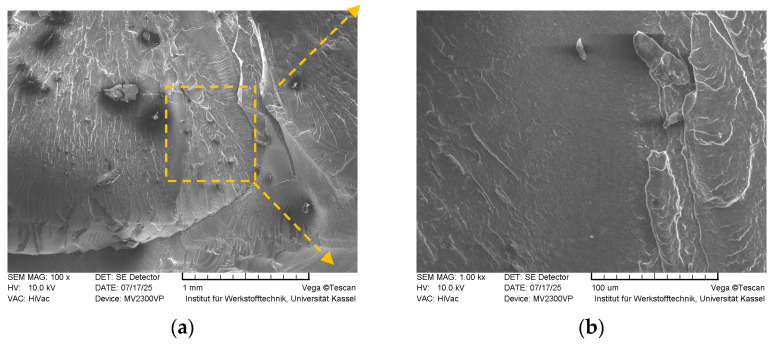
Fracture surfaces of the sample with *FF* = 1.21 acquired by SEM (magnified 100× (**a**), and 1000× (**b**), respectively).

Fracture surface SEM images of the sample with *FF* = 1.21 are seen in Figure 20.

The fracture morphology appears relatively smoother and flatter, suggesting a tendency toward brittle fracture or failure localized along a specific layer or fusion boundary. However, this sample exhibited the highest tensile strain and the second highest tensile stress among the tested specimens. These findings indicate that the mechanical properties improve as the overfilling phenomenon diminishes or ceases, highlighting the beneficial impact of reduced overfilling on the material’s structural integrity and deformation capacity.

**Figure 21:**
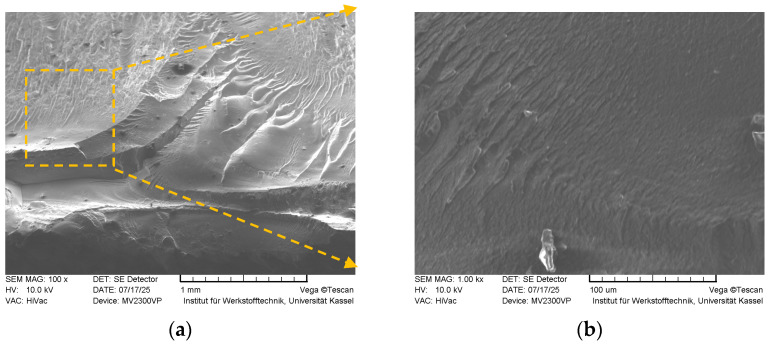
Fracture surface SEM images of the sample with *FF* = 1.24 (magnified 100× (**a**), and 1000× (**b**), respectively).

SEM images of the sample with *FF* = 1.24 are given in Figure 21. The fracture surfaces are notably smoother and more splayed compared to those of the *FF* = 1.16 and *FF* = 1.21 samples. The presence of fine lines, potentially identified as “river patterns,” indicates accelerated crack initiation and propagation. This morphological characteristic aligns with the tensile test results, which reveal a comparatively less ductile fracture behavior in this sample relative to the aforementioned specimens. Additionally, inter-layer cleavage observed on the surface may be associated with incomplete droplet coalescence, a feature corroborated by micro-computed tomography (µ-CT) imaging. This suggests that microscale defects at the layer interfaces contribute to the fracture mode and overall mechanical performance.

**Figure 22:**
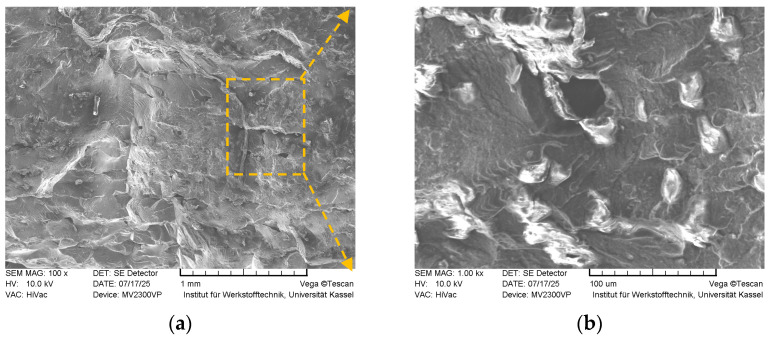
Fracture surfaces of the sample with *FF* = 1.28 acquired by SEM (magnified 100× (**a**), and 1000× (**b**), respectively).

Figure 22 depicts the fracture morphology of the sample with *FF* = 1.28.

Horizontal lines observed on the fracture surface indicate regional lack of fusion (LoF), which likely contributed to layer delamination and the presence of voids between deposited droplets. Within the context of the Arburg Plastic Freeforming (APF) process, these defects lead to structurally compromised parts characterized by internal discontinuities. Consequently, the tensile test results reflect this reduction in mechanical integrity. Therefore, it can be hypothesized that parts manufactured via the APF process with form factors exceeding 1.24 are unlikely to achieve satisfactory mechanical properties suitable for functional applications.

**Figure 23:**
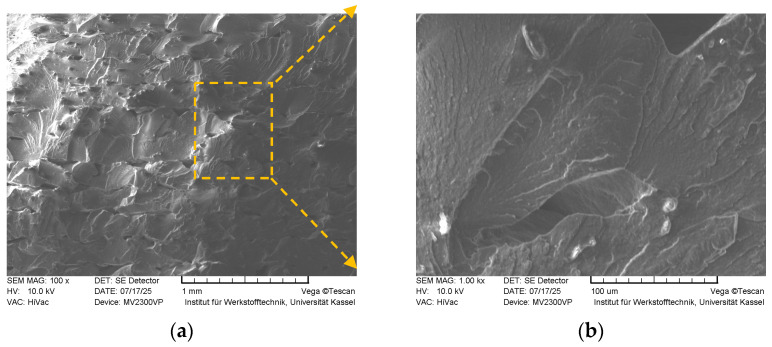
Fracture surface SEM images of the sample with *FF* = 1.31 (magnified 100× (**a**), and 1000× (**b**), respectively).

In Figure 23, fracture surfaces of the tensile sample with *FF* = 1.31 are given.

Similar to the observations for the *FF* = 1.28 sample, the fracture surface of the *FF* = 1.31 exhibits pronounced horizontal lines and voids indicative of lack of fusion (LoF) and delamination, with severity consistent with the tensile test results. These structural defects contribute to a more brittle fracture behavior, despite the sample achieving a tensile strain of approximately 6.43%, which exceeds the manufacturer’s specified minimum. Nonetheless, it can be inferred that the material’s load-bearing capacity was compromised by premature interlayer separation, limiting its overall mechanical performance.

### 3.5. Electrical Properties and Piezoelectric Behavior

**Figure 24:**
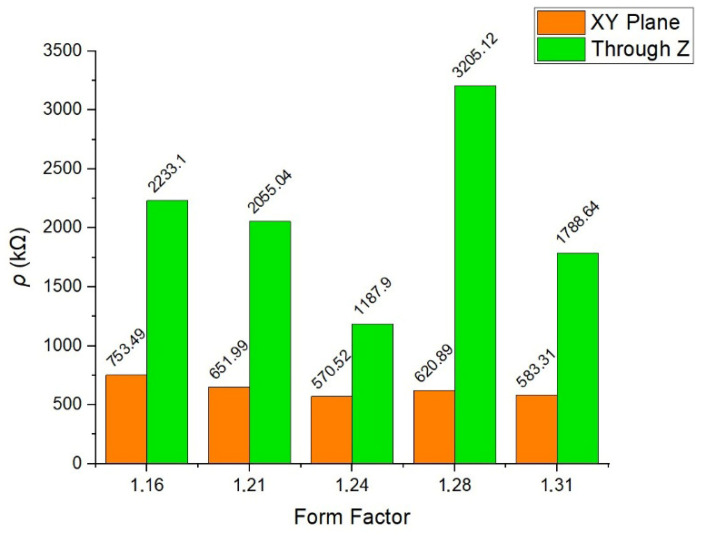
Resistivity of the cubic specimens through axial (XY) plane and *Z*-axis.

In Figure 24, the ascertained values of electrical resistivity of cubic samples are given.

As mentioned in Section 2.7, two sets of values that are measured both instantaneously and within 1 min period (after all waiting for up to 5 min to monitor even more cycles performed by the multimeter) are almost identical. A standard deviation of up to 1.01 for the results concerning XY plane is calculated, where it is dissimilar through *Z* axis (regarding XZ/YZ planes). Table 5 displays these values for all form factors included in the study.

A noteworthy increase in the resistivity is seen for the samples whose resistances are measured through the *Z* axis. For the sample *FF* = 1.31, a surprising decline in these values is noted; this specimen might have a better conductivity than *FF* = 1.28. Indeed, the standard deviation values of these sample sets are considerably high as related to the layered structure (through *Z* axis); thus, an inconsistency is still present for each of them.

**Figure 25:**
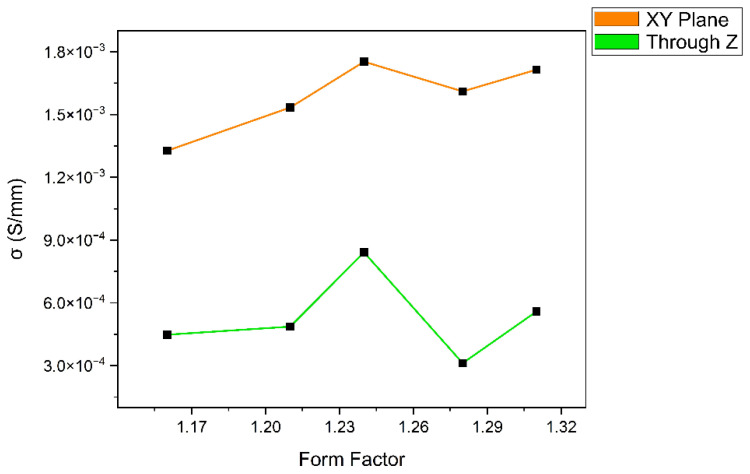
Conductivity of the cubic specimens through axial plane and *Z*-axis.

Calculated by using Equation (5), the conductivity of the samples is plotted as given in Figure 25.

Being the inverse of the resistivity, conductivity is clearly seen to increase as the form factor value raises up to 1.24. Afterwards a significant decline in it is observed with *FF* = 1.28, especially through the *Z* axis. Despite the fact that the conductivity steps up for *FF* = 1.31, the aforementioned standard deviation values of these samples (*FF* = 1.28 and the latter) are distinctly high, hence bringing their credibility into question.

**Figure 26:**
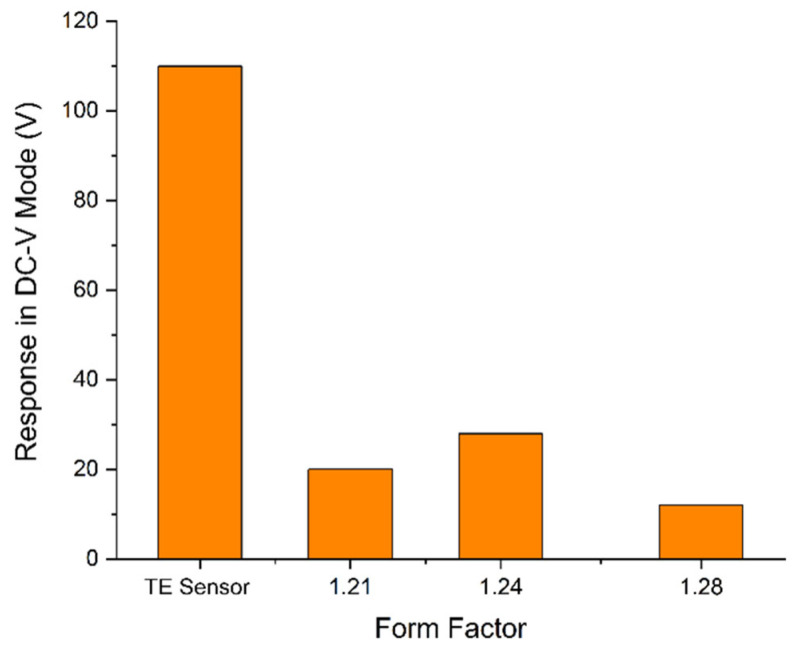
Circular single-layer samples’ ability to generate a piezoelectric-like potential difference, in comparison with the commercial sensor.

Concerning the piezoelectric behavior, the cubic specimens show imperfection as follows. During monitoring in the DC V mode of the multimeter, the measured values—for all form factors—after standardized impacts are 0.014 mV for XY plane, and 0.009 mV through *Z*-axis, respectively. To prevent any measurement errors and ensure repeatability, they are once more measured by switching to the resistance (Ω) mode of the multimeter, that yield the same, i.e., identical resistance values, which are already recorded. Afterwards the multimeter is switched to the DC V mode once more, and the impacts are delivered subsequently. Peculiarly, the acquired results do not change; only a slight difference is noticed for the sample with *FF* = 1.16 whose XY plane measurement yields between 0.008 mV and 0.009 mV, which should be negligible. For single-layer specimens, however, the findings are promising; although they make the multimeter show “over range” during the resistance (Ω) mode, once switching to the DC V mode and providing the ensuing 0.5 J impacts, the observed outputs are up to 28 V, as plotted in Figure 26. The conventional LDT0-028K sensor generates a piezoelectric-like potential difference of 110.49–110.62 V under the same conditions.

## 4. Discussion

The investigation into the APF processing of conductive PVDF reveals a critical dependency between the form factor, internal microstructural integrity, and the resulting mechanical as well as electrical performance. While previous studies on filament-based AM processes often suggest that maximum material density correlates directly with maximum strength, this study highlights a more complex relationship in droplet-based processing.

### 4.1. Correlation Between Form Factor and Porosity on the Basis of µ-CT

Providing a quantitative visualization of how *FF* dictates void formation, the µ-CT analysis emphasizes that there is a clear inverse correlation. As the *FF* increases, representing a lower discharge, i.e., overlap volume relative to the path spacing, porosity evolves from microscopic lack of fusion (LoF) voids to macroscopic structural gaps. The specimens with *FF* = 1.16 exhibited a near-fully dense structure (assuming <2% porosity), confirming that lower form factor values successfully force material into interstitial spaces. However, this high density comes at the cost of dimensional accuracy, resulting in the “protrusion” phenomenon [27]. By the same token, form factors above 1.28 result in distinct cross-hatch patterns and interconnected void networks, effectively transitioning the part from a solid to a mesh-like structure.

### 4.2. Mechanical Properties and Suitability of the Printed Parts for Real-Life Situations

The tensile test results demonstrate that the optimal mechanical window lies between the extremes of overfilling and under-extrusion. The highest tensile strengths (37.89 MPa and 38.66 MPa) were achieved at *FF* = 1.21 and 1.24, respectively, denoting that intermediate filling ratios may achieve optimal fusion of paths and minimal void content, thus also providing better strain thanks to the improved load transfer and delayed crack propagation. The following points are worth mention for a better understanding of the failure mechanisms.

- The overfilling penalty: It is notable that the densest sample (*FF* = 1.16) does not yield the highest mechanical strength. This suggests that excessive material deposition introduces internal residual stresses and potential over-compression of the droplets, which disrupts the load transfer mechanism (also the crystalline alignment that normally supports tensile deformation) despite the high density. This aligns with the SEM observations of rough fracture surfaces and plastic deformation, indicating that alongside the material yield, the structural integrity—particularly interlayer diffusion—is compromised by the printing pressure. At the bottom line, the material becomes more prone to premature fracture.
- The under-extrusion failure: At *FF* = 1.28, the drop in mechanical performance is precipitous. In particular the tensile strain dropped to 7.16%. This is directly supported by the SEM analysis, which reveals horizontal lines indicating LoF and layer delamination. The “river lines” observed in these samples suggest rapid crack propagation through these pre-existing void networks.

As the functional performance considering the dimensional accuracy of the printed parts is crucial for real-life applications, achieving a “viable” PVDF part requires balancing the need for fusion, i.e., density (while lightweight construction aspect is still in mind) against the preservation of geometric fidelity. The findings indicate that *FF* = 1.21 represents the optimal trade-off since it maintains sufficient overlapping for ductile failure modes, evidenced by relatively smooth morphology and high strain, while avoiding the severe warping and dimensional inaccuracies seen at *FF* = 1.16. Nevertheless, this form factor affects the part dimensions more or less (not as minimally as *FF* = 1.24), and the electrical performance accomplished with it is not as high as with *FF* = 1.24, which is discussed in Section 4.4.

Analogous to the FDM process, components manufactured by the APF method always exhibit microstructural porosity (inherently a certain void volume), characteristic of droplet-based deposition. While adjacent rounded tracks are to fuse through neck growth in between the tracks, solidification of the material prior to complete coalescence results in partial, i.e., residual inter-track voids [119,120]. Attempts to eliminate these voids by increasing the discharge rate (DR) will not fill them, leading admittedly to overfilling that is procedurally unacceptable, as it compromises the dimensional accuracy of the final part [27].

### 4.3. Possible Strategies to Overcome the Defects Perceived by SEM Analysis

Despite their good ductility, the pronounced roughness and tear ridges of *FF* = 1.16 and *FF* = 1.21 indicate excessive internal stress and/or material pushing out sideways. Since these samples also suffer from being outside tolerance, i.e., oversized, a strategy such as reducing the flow rate slightly could move the parameters closer to the state of *FF* = 1.21, hence mitigating the overfilling roughness.

*FF* = 1.24: To overcome the tendency towards brittle fracture due to the “river patterns”, believed to initiate rapid crack formation and propagation at the layer interfaces, increasing the overlapping of droplets may be a good strategy to remove the voids that act as stress concentrators. With as many voids dispelled, the material should yield rather than snap, exhibiting a more ductile behavior seen in the lower form factors. However, this approach requires either increasing the DR [27] or varying the pulsing frequency of the piezoelectric actuator (by increasing the volume of individual droplets), and is out of the question where only the influence of different form factors is investigated. Reducing the spacing of droplets can be accomplished not only by lowering the form factor, but also by increasing the nozzle temperature; it is believed that incomplete droplet coalescence occurs when the material viscosity is too high upon deposition, and a higher nozzle temperature lowers viscosity slowly, thus allowing the droplets to fuse better before solidifying. On the downside, the risk of over-extrusion comes into play, which is reported to result in poor print quality and surface imperfections [121]. Alternatively, reducing the printing speed might help while maintaining the same temperature.

Being a classic Z-strength issue (in other words poor Z-direction performance) in additive manufacturing, the delamination/premature interlayer separation due to the regional lack of fusion seen at *FF* = 1.28 and higher may be alleviated by, e.g., reducing layer thickness [122] or raising the chamber temperature. The first action may promote better diffusion in between layers as the nozzle is forced closer to the previous printed layer (as increasing the pressure during deposition), yet the latter is also questionable with the Curie temperature of PVDF in mind even though the already printed, i.e., previous layer remains more receptive to bonding.

Table 6 summarizes these strategies and why they might not be directly applicable.

### 4.4. Customization Potential of the Printed Parts for Different Applications Regarding Electroactivity

The research data suggest a divergence between the mechanical optimum (*FF* = 1.21) and the “functional” optimum in terms of electrical function (*FF* = 1.24). While the samples with *FF* = 1.21 are structurally dense and mechanically strong, they are functionally inferior as compared to the ones with *FF* = 1.24, for this form factor yields the best conductivity/piezoelectric response. On the other hand, the specimens printed at *FF* = 1.24 and higher are mechanically on the edge due to the fact that they show river patterns and incipient interlayer cleavages if exposed to load, even regional lack of fusion (LoF) at *FF* = 1.28. It must also be taken into account that the slight dimensional inaccuracies observed in samples with *FF* = 1.21 and *FF* = 1.28 (yet completely opposite of each other) by means of numerous measurements, such as µ-CT, are not desired in practice; geometric fidelity is favored not only for rapid prototyping/tooling, but also for end parts and interlocking layers, i.e., components which can be successfully produced thanks to the multi-material capabilities of the Freeformer.

The negligible variance between instantaneous measurements and those taken after a 1 min (moreover up to 5 min.) interval could indicate that the samples exhibit a stable electrical response without significant capacitive charging effects or thermal drift during the testing frame. This establishes the reliability of the data acquisition method utilized in Section 2.7. A pronounced anisotropy is observed when comparing the axial (XY) plane to the *Z*-axis. The XY plane results demonstrate high consistency with a low standard deviation (*σ*≈1.01). In contrast, measurements taken through the *Z*-axis (XZ/YZ planes) exhibit significantly higher resistivity, with a notably higher standard deviation especially for the samples with *FF* = 1.28 and *FF* = 1.31. This behavior is attributed to the characteristics of additive manufacturing, as it is believed that the interfaces between deposited layers act as potential barriers to electron flow. The increased resistivity in the Z-direction can similarly be attributed to contact resistance at these inter-layer boundaries, which is less prevalent in the continuous deposition paths of the XY plane.

Beginning with *FF* = 1.16, an inverse correlation between conductivity and form factor is evident as the *FF* increases to 1.24. Although the increase in material discharge ratio should improve the coalescence of droplets, reducing void volume and creating more continuous conductive pathways, the overfilled samples (*FF* = 1.16 and *FF* = 1.21, respectively) do not exhibit as high conductivity as the ones with *FF* = 1.24. Along with the lack of fusion (LoF), surface roughness and/or geometrical inaccuracies might be the reason for the significant decline in conductivity (as well as piezoelectric performance) of *FF* = 1.28, having disrupted the contact surface for measurement or the internal continuity of the layers. While the sample set with *FF* = 1.31 indicates a potential recovery in conductivity, the reliability of these results is compromised by a high standard deviation. The distinct inconsistency in the *Z*-axis values for these samples implies that higher form factors (≥1.28) may induce irregular layer stacking or variable surface topology, resulting in unpredictable electrical performance.

In view of these statements, interlayer contact resistance as well as structural anisotropy are figured to be the reason for the imperfect piezoelectric response of multi-layer (solid) specimens, apart from the single-layer ones. It is still arguable to attribute the XY plane and/or *Z*-axis findings, i.e., results to the percolation theory for any sample sets, since it must be confirmed if the changing form factor causes the conductive particles to be pressed closer together, re-establishing a percolation network that might be broken by voids; comprising a non-conductive (pure) PVDF film, the commercial reference sensor yields peak open-circuit voltages over 110 V, while the single-layer conductive specimens exhibit distinct peak responses, yet under 30 V. This attenuation in voltage magnitude may be attributed to the presence of the conductive carbon network, on which it is conjectured that the internal impedance of the material is reduced; this network for charge transport could act as a leakage path for the generated charge, thereby diminishing the maximum achievable potential difference. With respect to the higher output of *FF* = 1.24, it can be considered for the lower form factors (here *FF* = 1.21) that the overfilling often introduces internal stress or surface irregularities that can disrupt the polarization alignment and/or create defects, which lead to a drop in performance despite having more material in the (micro)structure. For higher form factors, however, microscopic voids between droplets, i.e., insufficient overlapping might fail to accomplish the mechanical transmission of the impact force to the PVDF crystalline domains (as with *FF* = 1.28), hence generating lower voltage. As the reference sensor is a near-perfect dielectric (insulator), it is debatable whether the conductive specimens perform like a resistor to some degree; thus, the charge leaks faster than it can ascend up to 100+ Volts. Whilst taking the already performed characterization methods and reported values in the literature [65,66,67,68,69,70], using an oscilloscope to obtain, e.g., decay curves during the impacts as well as investigating piezoresistive effect/sensitivity [123] in this grade of PVDF should set the stage for additional research; nonetheless, the scope of this study accounts for the influence of the form factor and initial endeavor to 3D-print functional layers/components in lieu of ascertaining advanced features of the material.

**Figure 27:**
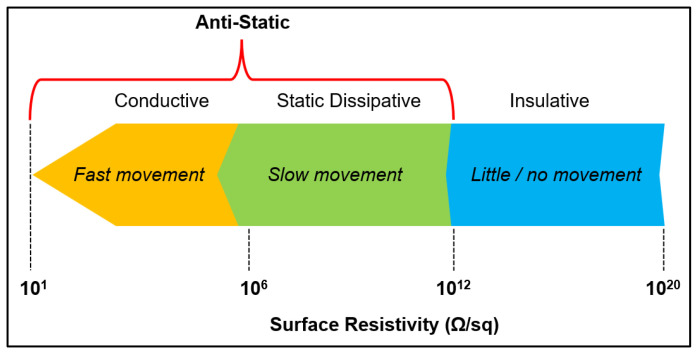
ESD range related to the movement of electrons and the materials’ surface resistivity.

For all that, multilayer PVDF parts are to be employed as conductive elements (rather than ESD materials thanks to their higher conductivity, as well as the material’s electromagnetic interference/EMI shielding performance and even fulfilling ATEX requirements [124] where required), although they cannot be intended for sensor or functional surface applications like the single-layer ones. Figure 27 provides insight into the electrically conductive spectrum regarding electron movement [125].

Furthermore, PVDF layers or modules may be designated for being the functional surfaces/elements in a multi-material 3D-printed unit (as proposed in the introduction section) where the main material is different, yet a compatible one: a prosthetic hand of TPU or low-cost ABS material with PVDF inlays/sensors to help process tactile feedback, i.e., stimuli, or a simple (preferably single-layer) haptic trackpad/touchpad in a frame made of TPU or Poly(methyl methacrylate) (PMMA) for, e.g., automotive applications. More examples like these could be projected. It is important to bear in mind that in addition to its ascertained dimensional accuracy, the form factor *FF* = 1.24 is desirable since the samples produced using this *FF* value demonstrate superior electrical conductivity and piezoelectric behavior. In this respect it is presumed that the slightly reduced material deposition rate (compared to *FF* = 1.16 and *FF* = 1.21) facilitates the alignment of dipoles or the formation of the electroactive β phase, potentially due to favorable shear stress conditions or thermal histories coherent to this parameter set, thus urging the need for further research.

## 5. Conclusions and Future Outlook

This study successfully demonstrates the processability of electrically conductive carbon-reinforced PVDF utilizing the Arburg Plastic Freeforming (APF) process, and systematically evaluates the influence of the form factor (*FF*) on the microstructural integrity, mechanical performance, and electroactive response of the printed parts with different geometries. By the comprehensive analysis of porosity via µ-CT, correlated with tensile testing and characterization of fracture surfaces per SEM, eventually following the final verification of the observed conductive and piezoelectric behavior, the following conclusions are drawn.

1. Optimal processing window based on *FF*: The optimal form factor for this specific grade of PVDF in the APF process is identified between 1.21 and 1.24. This range yields the highest tensile strength (~38 MPa) and strain-at-break (~11–12%), significantly outperforming both over-extruded and under-extruded samples. While lower form factors (*FF* = 1.16) result in overfilled, dense structures characterized by ductile failure and significant plastic deformation, higher form factors exceeding 1.28 lead conversely to substantial underfilling, manifested as regional lack of fusion (LoF) and void formation, which severely compromises the structural integrity and caused premature delamination.
2. Microstructural influence: µ-CT analysis and SEM characterization confirm that porosity is the governing factor for mechanical failure especially in higher form factors (>1.28). Large interconnected voids act as stress concentrators, leading to brittle delamination, as confirmed in particular by SEM.
3. Dimensional accuracy: While lower form factors (<1.24) maximize density, they compromise the dimensional accuracy of the component due to material overflow and accumulation. Higher form factors, in an analogous manner, cause a slight concavity in at least one of the planes, thus negatively affecting geometric fidelity.
4. Electroactive Capabilities: Although the structural and mechanical properties of the 3D-printed PVDF are of high importance in the field, the material’s intrinsic value lies in its individual features, such as electroactive capabilities. It is proven that the APF process does not negatively affect the conductivity of the material in this sense (regarding manufacturer’s declaration as well); however, single-layer prints have a much better potential than multilayer, i.e., solid produced parts for applications that require piezoelectric response rather than solely conductivity.

In light of these conclusions, the following key points are highlighted as the future outlook.

- Narrowing down the *FF* window: Specifying the processing range between 1.21 and 1.24, within the bounds of possibility, i.e., at intervals of 0.01 or even smaller to investigate the effects further.
- Piezoelectric activation: Specifically, the influence of thermal or corona poling on the ß-phase content of the APF-processed parts may be investigated by, e.g., Fourier transform infrared spectroscopy (FTIR), and these methods can be—during or after printing—realized to maximize the piezoelectric coefficient (*d*_33_). Capacitance measurements and validation of *d*_33_ values, using, e.g., a wide-range *d*_33_ tester or a Berlincourt quasi static piezoelectric *d*_33_ m, are considered in this sense as future work.
- Thermal post-processing: Additional scrutiny into post-printing annealing could be conducted to relieve the residual stresses observed in the overfilled samples, potentially allowing for higher-density parts with improved mechanical ductility. Methods such as thermal contact poling as mentioned above should also be expedient to evolve the electrical, i.e., piezoelectric performance; however, the applied temperature range during the process must be taken into account if the part consists of multiple materials (as per the multi-material 3D-printed PVDF-TPU and PVDF-PMMA examples given in Section 4.4).
- Sensor applications: Given the successful printing of thin, single-layer specimens in this study, the next logical step can be the development of functional prototypes, such as tactile sensors or energy harvesters, utilizing the conductive nature of the carbon-based reinforcement as integrated electrodes likewise. Self-sensing power electronics enclosures and battery boxes for, e.g., electric vehicles (EVs), are also aimed to be designed and developed; however, manufacturing the whole component from PVDF might not be sustainable in regards to the material availability and prices apart from the difficulties of processing the material and its composites.

In summary, it is confirmed that the material’s conductive integrity is preserved during processing, and furthermore, there is a distinct advantage for single-layer structures in piezoelectric applications compared to solid, multilayer builds. Therefore, this sets the stage for future investigations into detailed process characterization and functional optimization, specifically through fine-tuning processing intervals, implementing thermal and corona poling strategies—where practicable—to enhance electromechanical coupling, and ultimately translating these findings into the development of 3D-printed tactile sensors and energy harvesting components.

## Figures and Tables

**Table 1:** Typical properties of Arkema Kynar^®^ 340 PVDF [77].

	Value	Unit	Test Standard
Density	1.78	g/cm^3^	ISO 1183 [90]
Melt flow rate (MFI)	3.0–8.0	g/10 min	ASTM D1238 [91]
Shore hardness	76	D	ISO 868 [92]
Melting point, 73 °F (~23 °C)	165–172	°C	ASTM D3418 [93]
Volume resistivity (approx.)	10^4^	Ohm×m	IEC 62631-3-1 [94]
Surface resistivity (approx.)	10^4^	Ohm	IEC 62631-3-2 [95]

**Table 2:** Form factor values of sample sets that are subjected to further investigation.

Form Factor	Cubic	Circular
1.16	+	−
1.21	+	+
1.24	+	+
1.28	+	+
1.31	+	−

**Table 3:** Machine parameters for manufacturing the specimens.

Parameter	Value	Unit
Nozzle temperature	255	°C
Temperature of heating zone 2	235	°C
Temperature of heating zone 1	220	°C
Chamber temperature	90	°C
Printing speed of outer walls	30	mm/s
Printing speed of infill	75	mm/s
Discharge rate	70	-

**Table 4:** Mechanical properties of samples including standard deviation values.

Form Factor	Ultimate Strength (MPa)	Strain (%)	Elongation at Break (mm)
1.16	36.46 ± 2.49	11.51 ± 0.78	5.75 ± 0.78
1.21	37.89 ± 1.32	12.87 ± 0.72	6.43 ± 0.72
1.24	38.66 ± 0.45	10.86 ± 0.73	5.43 ± 0.73
1.28	36.46 ± 0.85	7.16 ± 0.55	3.58 ± 0.55
1.31	34.12 ± 0.49	6.43 ± 0.35	3.21 ± 0.35

**Table 5:** Standard deviation values calculated using the results of electrical resistivity measurements.

Form Factor	SD for XY Plane	SD for *Z* Axis
1.16	1.011	2.545
1.21	0.261	0.014
1.24	0.388	0.622
1.28	0.856	6.236
1.31	0.558	21.679

**Table 6:** Possible procedures to overcome structural defects and their applicability.

Observed Defect (SEM)	Associated *FF* Range	Suspected Root Cause	Mitigation Approach	Practicality
Tear ridges and roughness	Low (1.16–1.21)	Overfilling (excess material overlapping)	Reducing flow rate, decreasing discharge rate (DR)	The latter is ineligible since it may cause under-extrusion and/or dimensional inaccuracy.
Interlayer cleavage: “river patterns”	Medium (1.24)	Accelerated crack initiation from micro-voids	Improving droplet coalescence via thermal management e.g., increasing nozzle temperature	Reducing the printing speed may be a better course of action not to cause the material deteriorate and/or over-extruded.
Lack of fusion (LoF)	High (1.28–1.31)	Insufficient droplet overlap	Reducing layer thickness, increasing DR	Further investigation with new parameter set needed
Layer delamination	High (1.28–1.31)	Poor thermal bonding in-between layers	Reducing layer thickness, increasing chamber temperature	The latter is disputable with respect to the Curie Temperature of PVDF.

## Data Availability

The original contributions presented in the study are included in the article, further inquiries can be directed to the corresponding author.

## Abbreviations

The following abbreviations are used in this manuscript:

APF	Arburg Plastic Freeforming
PVDF	Polyvinylidene Fluoride
µ-CT	X-ray computed microtomography
SEM	Scanning electron microscope
AM	Additive manufacturing
FDM	Fused deposition modeling
FGF	Fused granulate fabrication
MEX	Material extrusion
PEM	Pellet extrusion modeling
ABS	Acrylonitrile butadiene styrene
DAR	Drop aspect ratio
TrFE	Trifluoroethylene
TFE	Tetraflouoroethylene
MWCNT	Multi-walled carbon nanotube
EAP	Electro-active polymer
*T_C_*	Curie temperature
TPU	Thermoplastic polyurethane
ESD	Electrostatic discharge
HT	High-temperature
DR	Discharge rate
PC	Polycarbonate
CAD	Computer-aided design
DIN	Deutsches Institut für Normung—German Institute for Standardisation
EN	Europaische Norm—European standard
ISO	International Organization for Standardization
SE	Secondary electron
SI	Système international d’unités—International System of Units
ASTM	American Society for Testing and Materials
PLA	Polylactic acid
LoF	Lack of fusion
DC	Direct current
V	Volt
EMI	Electromagnetic interference
ATEX	Atmosphères explosives—explosive atmospheres
PMMA	Poly(methyl methacrylate)
*d*_33_	Piezoelectric coefficient
EV	Electric vehicle
